# Meta-Analysis of Microarray Data Identifies *GAS6* Expression as an Independent Predictor of Poor Survival in Ovarian Cancer

**DOI:** 10.1155/2013/238284

**Published:** 2013-06-27

**Authors:** Michelle Buehler, Brian Tse, Alix Leboucq, Francis Jacob, Rosmarie Caduff, Daniel Fink, Darlene R. Goldstein, Viola Heinzelmann-Schwarz

**Affiliations:** ^1^Translational Research Group, University Hospital Zurich, 8091 Zurich, Switzerland; ^2^Ovarian Cancer Group, Adult Cancer Program, Lowy Cancer Research Centre, Prince of Wales and School of Women's and Children's Health Clinical School, University of New South Wales, Sydney, NSW 2052, Australia; ^3^Mathematics Institute for Analysis and Applications (MATHAA), EPFL, 1015 Lausanne, Switzerland; ^4^Gynecological Research Group, University Hospital Basel, 4031 Basel, Switzerland; ^5^Institute of Clinical Pathology, University Hospital Zurich, 8091 Zurich, Switzerland; ^6^Department of Gynecology, University Hospital Zurich, 8091 Zurich, Switzerland

## Abstract

Seeking new biomarkers for epithelial ovarian cancer, the fifth most common cause of death from all cancers in women and the leading cause of death from gynaecological malignancies, we performed a meta-analysis of three independent studies and compared the results in regard to clinicopathological parameters. This analysis revealed that *GAS6* was highly expressed in ovarian cancer and therefore was selected as our candidate of choice. *GAS6* encodes a secreted protein involved in physiological processes including cell proliferation, chemotaxis, and cell survival. We performed immunohistochemistry on various ovarian cancer tissues and found that GAS6 expression was elevated in tumour tissue samples compared to healthy control samples (*P* < 0.0001). In addition, GAS6 expression was also higher in tumours from patients with residual disease compared to those without. Our data propose GAS6 as an independent predictor of poor survival, suggesting GAS6, both on the mRNA and on the protein level, as a potential biomarker for ovarian cancer. In clinical practice, the staining of a tumour biopsy for GAS6 may be useful to assess cancer prognosis and/or to monitor disease progression.

## 1. Introduction

Epithelial ovarian cancer (EOC) is the fifth most common cause of death from all cancers in women and the leading cause of death from gynecological malignancies [1, 2]. Most patients (70%) present at initial diagnosis with locally advanced or disseminated disease, which is characterized by invasion of surrounding organs and in high stage cases of the peritoneal cavity. The survival rate of women with widespread metastatic disease is a dismal 10–20% [3]. This poor overall prognosis is due to the lack of screening tools for early stage disease, the nonspecific nature of symptoms, and drug resistance in advanced disease. A major challenge is the identification of new tumour markers. These will improve diagnosis and may serve as prognostic indicators and targets for new therapeutic strategies [4].

Cancer biomarkers can exist in various forms among others as DNA (genome), mRNA (transcriptome), cell surface or secreted proteins (proteome), and carbohydrates (glycome). Transcriptomic microarrays provide a broad picture of gene expression by monitoring the intensity expression levels of thousands of genes simultaneously, which together provide a molecular blueprint of the tumours, or what is defined as an expression profile [5]. The use of transcriptomic-based high-throughput platforms is therefore a good starting point for the identification of cancer-relevant biomolecules. However, most studies are based on a single patient cohort and often have small sample size. Meta-analysis of transcriptomic data from several independent studies is a powerful approach to detect biomarkers with much greater sensitivity and, potentially, specificity.

 In order to identify biomarkers for ovarian cancer based on survival, we performed a meta-analysis of independent studies based on individual observations [6–9]. From this analysis *GAS6 *emerged as our candidate of choice. *GAS6* is overexpressed in various cancers including ovarian cancer [10–12]. Its encoded product GAS6 is a secreted protein involved in a broad range of physiological processes including the induction of cell proliferation, chemotaxis, and survival [13–15]. To the best of our knowledge, there has only been one other study that investigated GAS6 expression in ovarian cancer [16]. The authors observed that *GAS6* and its encoded protein were overexpressed in ovarian cancers; however, the relationship between GAS6 expression in levels and various clinicopathological parameters was not reported. In our study with a large cohort of healthy controls and adenocarcinoma patients, immunohistochemistry for GAS6 expression in epithelial ovarian cancer samples confirmed the findings of our meta-analysis; comparison of the data with various clinicopathological parameters identified *GAS6* as an independent predictor of poor prognosis.

## 2. Materials and Methods

### 2.1. Meta-Analysis

Combining raw data from microarray studies on different platforms remains problematic due to data that are not commensurable. Meta-analysis of test statistics from different studies provides a powerful and robust way to integrate heterogeneous microarray studies. We carried out a meta-analysis of initially four similarly designed microarray studies of ovarian cancer [6–9]. After the retraction of the Dressman paper [9] in February 2012, we recalculated the meta-analysis of the three remaining studies.

 We used the analysis strategy outlined in Wirapati et al. [16]. Beginning with the complete preprocessed primary data, probes were matched to UniGene identifiers. We considered the union of all genes that are represented in at least one study. For each gene, we computed a normally distributed test statistic measuring the association of expression with presence of ovarian cancer (probit-transformed *t*-statistic). We then meta-analytically combined these single gene individual study *z*-scores *Z*_*ik*_ across studies using equal weighting by the inverse normal method [17]:

(1) $$\begin{array}{c} \bar{Z_{i}}=\sum_{k=1}^{K_{i}}\frac{Z_{ik}}{\sqrt{K_{i}}}, \end{array}$$

where *i* indicates gene, *k* indicates study, and *K*_*i*_ is the number of datasets where gene *i* is present (i.e., any platform missing the gene is ignored). The resulting $\bar{Z_{i}}$ should all be (approximately) distributed as standard normal and are ranked according to size (or, equivalently, by *P* value).

### 2.2. Clinicopathological Patient Cohort

Two patient cohorts from the University Hospital Zurich and Spital Limmattal were chosen for this study: (a) prospectively included patients prior to surgery for unknown pelvic mass after giving informed consent in accordance with ethical regulations (SPUK, Canton of Zurich, Switzerland; StV Nr. 06/2006), and (b) ovarian tumour patients diagnosed since 1991 which were retrospectively included after receiving ethical allowance. Patients with a history of cancer or autoimmune diseases were excluded. The cohort consisted of 800 patients and was composed of three major patient groups: (1) healthy patients with normal ovaries and tubes; (2) benign tumours or borderline tumours of the ovaries; (3) epithelial ovarian, tubal, or peritoneal cancers. All clinicopathological patient data such as FIGO stage, grade, residual disease, presence of ascites, past and present medical illness, ultrasonic findings and outcome data were stored in a specially designed in-house database (PEROV) based on ACCESS (Microsoft, USA). Histopathology of all study patients was independently re-evaluated by a pathologist specialized in the field of gynaecological oncology (R.C.), and patients with unclear or mixed diagnoses were excluded from the study. Tissue microarrays were constructed using a Tissue Microarrayer (Beecher Instruments, USA). Each patient was represented by two cores (1.0 or 2.0 mm) from different regions of the tumour.

### 2.3. Immunohistochemistry

For the detection of GAS6, tissue microarray slides were stained using the Ventana Benchmark automated staining system (Ventana Medical Systems, USA). For antigen retrieval, slides were incubated with Tris-based buffer (with slightly alkaline pH) for 1 hour, according to standard procedures. Slides were incubated with the anti-human GAS6 (R&D Systems, USA) for 1 hour. Negative controls involved the omission of the primary antibody. Counterstaining was performed with hematoxylin and 1% acid alcohol. To assess GAS6 expression level, a weighted average (WA) method of scoring was performed. First, each core sample was scored for overall percentage (between 0 and 100) and overall intensity (0 to 3). The overall percentage and overall intensity of each sample were then multiplied, with the resultant number for each sample core within the same cohort summed, and divided by the total number of cores. Scoring was independently assessed by a researcher (M.B.) and a pathologist (R.C.), and discrepancies were resolved by consensus.

### 2.4. Cell Lines and Tissue Culture

The human ovarian cancer cell lines IGROV1, SKOV3, OVCAR3, and A2780 (all serous) were maintained in RPMI media, and TOV112D (endometrioid) and TOV21G (clear cell) cell lines were cultured in DMEM media. The human ovarian surface epithelial cell lines HOSE6.3 and HOSE17.1 were cultured in a 50 : 50 mix of Media 199 and MCDB 105 media. All culture media were supplemented with 10% foetal bovine serum, 2 mM L-glutamine, and penicillin/streptomycin. Cells were incubated at 37°C in a humidified atmosphere of 5% CO_2_/air.

### 2.5. Western Blot

Subconfluent cells were washed thrice in PBS, then scraped off flasks in RIPA buffer (150 mmol/L NaCl, 1% Triton X-100, 0.1% SDS, and 25 mmol/L Tris-Cl (pH 7.5)), and supplemented with protease inhibitor cocktail tablets (Roche Diagnostics, Germany). The cells were snap-frozen in liquid nitrogen, thawed, and then centrifuged at 13,000 g for 10 mins. The supernatant (whole cell lysate) was quantified using the BCA protein estimation reagent (Thermoscientific, USA). Twenty micrograms of lysate was mixed with NuPAGE loading dye and reducing agents (Invitrogen) then heated at 95°C for 5 mins before loading onto sodium dodecyl sulphate (SDS)-polyacrylamide gels (Invitrogen). All gels were transferred to PVDF membranes and blocked for 1 hour at PBS containing 0.1% Tween 20 (PBS/T20). Membranes were incubated with the primary antibodies for GAS6 (anti-human GAS6; R&D Systems, USA) and GAPDH (anti-human GAPDH; Santa Cruz, USA) in PBS/T20 overnight at 4°C, washed thrice with PBS/T20, and incubated with secondary antibodies conjugated to horse radish peroxidase (rabbit anti-goat IgG-HRP; Santa Cruz, USA). Membranes were washed again in PBS/T20, incubated with ECL (Elmer Perkins, Australia), and then developed on photo film (Sigma, USA). Quantification of signal intensities was performed using Bio-Rad GS-800 densitometer with Quantity One software (Hercules, USA).

### 2.6. Statistical Analysis

Immunohistochemical scoring as assessed by intensity and percentage of stained cells was combined in a weighted average score, which takes in account both values and the number of cores available per patient: ((percentage 1 × intensity 1) × (percentage *n*× intensity *n*)/number of cores). Box plots were created for weighted average values depending on the clinicopathological diagnosis. To see whether there is a difference in means between those groups, an analysis of variance (ANOVA) was performed. All western blot experiments have been performed as triplicates (B.T.). Comparison of two data values was measured for their significance using Mann-Whitney *U* tests; in relation to multiple values, the Kruskall-Wallis test was applied (a nonparametric method for testing differences in median between several groups). Correlations of clinicopathological data with outcome parameters were performed using the Kaplan-Meier analysis (univariate analysis) and multivariate using Cox proportional hazard models. Meta-analysis and all statistical analyses have been performed by two expert mathematicians specialising in the analyses of bioinformatics high-throughput data analysis (D.G., A.L.). All data analysis except the meta-analysis were performed using the open source statistical programming language R (http://CRAN.R-project.org/, version 2,8.1). A *P* value of <0.05 was considered as statistically significant.

## 3. Results

### 3.1. Characteristics of Patient Cohort

Our cohort consisted of a variety of tissues: epithelial ovarian cancers (*n* = 172), ovarian borderline (*n* = 40), cystic adenoma (*n* = 2), normal fallopian tubes (*n* = 3), and ovarian surface epithelium (*n* = 6). In the cancer cohort of 172, 18.6% were of FIGO stage I, 9.9% of stage II, 41.9% of stage III, and 25% of stage IV. The predominant histotype was serous (48.8%), followed by clear cell (24.4%), then endometrioid (22.7%), and transitional (4.1%). Fifty-six percent of women were over the age of 60, compared to 43.6% under 60. Other clinicopathological parameters accessible within our cohort included TNM-T, TMN-N, and TMN-M scores, grade, the presence of ascites at initial diagnosis, primary cancer origin, overall health, preoperative CA125 levels, relapse, sensitivity to platinum chemotherapy, and progressive disease (Table 1).

### 3.2. Meta-Analysis of DNA Microarray from Four Publications Identifies *GAS6* as a Highly Ranked Robust Candidate Biomarker for Ovarian Cancer

Although no single study shows *GAS6* to rank particularly high, meta-analysis identifies *GAS6* as an interesting potential biomarker. *GAS6* ranks in the middle of the top candidates (combined *z* = 3.10, nominal *P* value < 0.001) when considering both DS and *z*-scores genes. Several other highly ranked genes have achieved their rank based on one outlier *z*-score. However, the *GAS6* result is robust when considering not only the combined *z*-score in isolation but also the variability of the individual *z*-scores. This is shown in Table 2 as well as in a reproducibility plot (data not shown) incorporating both the standard deviation (SD) and the combined *z*-score of all 4 transcriptomic data sets. The highly upregulated ovarian cancer genes from this meta-analysis are listed in Table 2 in the order of increasing SD (decreasing reproducibility). Hereby, *GAS6* is roughly in the middle of this top candidate group, where also well-known ovarian cancer markers like HE4 (WFDC2) or DDR1 are listed. Moreover, 50% (17/34) of these top candidates have been linked to ovarian cancer in the scientific literature. These data suggest a high degree of consistency across the diverse studies and confirming its suitability as a biomarker. 

### 3.3. GAS6 Is Overexpressed in Ovarian Cancers

**Figure 1:**
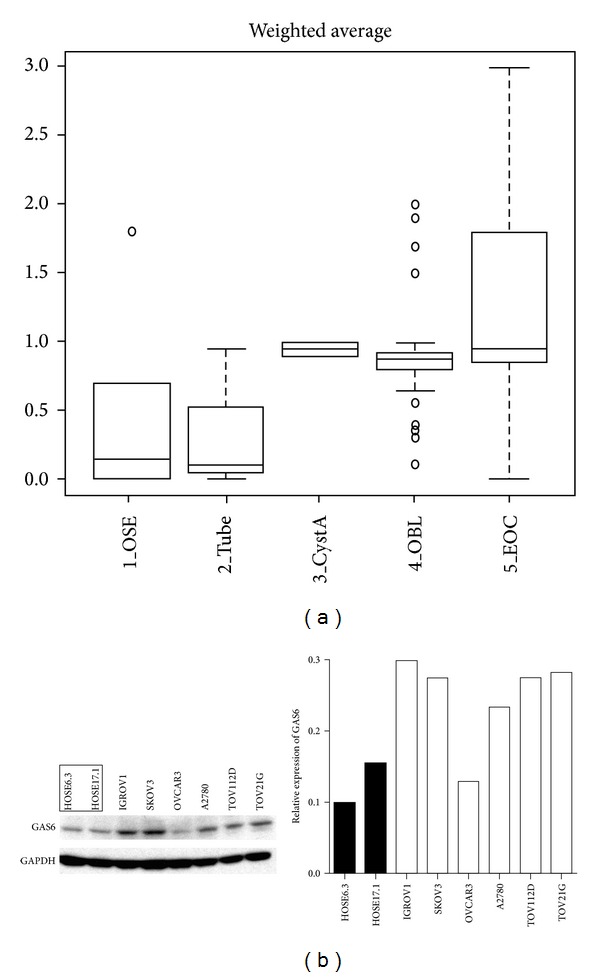
GAS6 is overexpressed in ovarian cancers. (a) Box plot demonstrating GAS6 weighted average expression scores on various ovarian tissues as assessed by immunohistochemistry. Statistical test performed was the Kruskal-Wallis test. OSE: ovarian surface epithelium; CystA: cystadenoma; OBL: ovarian borderline tumour; EOC: epithelial ovarian cancer. (b) Western blots for GAS6 on a panel of ovarian cell lines. Left panel shows original blot, right panel shows relative expression based on densitometric analysis (density of GAS6 divided by density of GAPDH). Results from a representative experiment are shown. Normal cell lines (HOSE6.3 and HOSE17.1) are marked (framed or black bars).

With our meta-analysis of transcriptomic data revealing that *GAS6* mRNA is highly expressed in ovarian cancer, we assessed the expression of GAS6 in various ovarian tissues by immunohistochemistry. Strong cytoplasmic staining of GAS6 was observed in epithelial ovarian cancer tissues. Based on the weighted average (WA) method of scoring, GAS6 expression was stronger in epithelial ovarian cancers (EOC; *n* = 172) and ovarian borderline tumours (OBL; *n* = 40) than in ovarian surface epithelium (OSE; *n* = 6) or normal fallopian tubes (Tube; *n* = 3) (*P* = 4.97 × 10^−5^) (Figure 1(a)). This trend of greater expression in tumour than in normal tissue was confirmed by western blot on whole cell lysates of a panel of human ovarian cell lines where, apart from the OVCAR3 cell line, all the ovarian carcinoma cell lines IGROV1, SKOV3, A2780 (all serous), and the TOV112D (endometrioid) and the TOV21G (clear cell) cell lines displayed elevated GAS6 expression compared to the two human ovarian epithelial cell lines (nontumour origin) HOSE6.3 and HOSE17.1 (Figure 1(b)).

### 3.4. GAS6 Expression Is Elevated in Ovarian Cancers from Patients with Residual Disease

**Figure 2:**
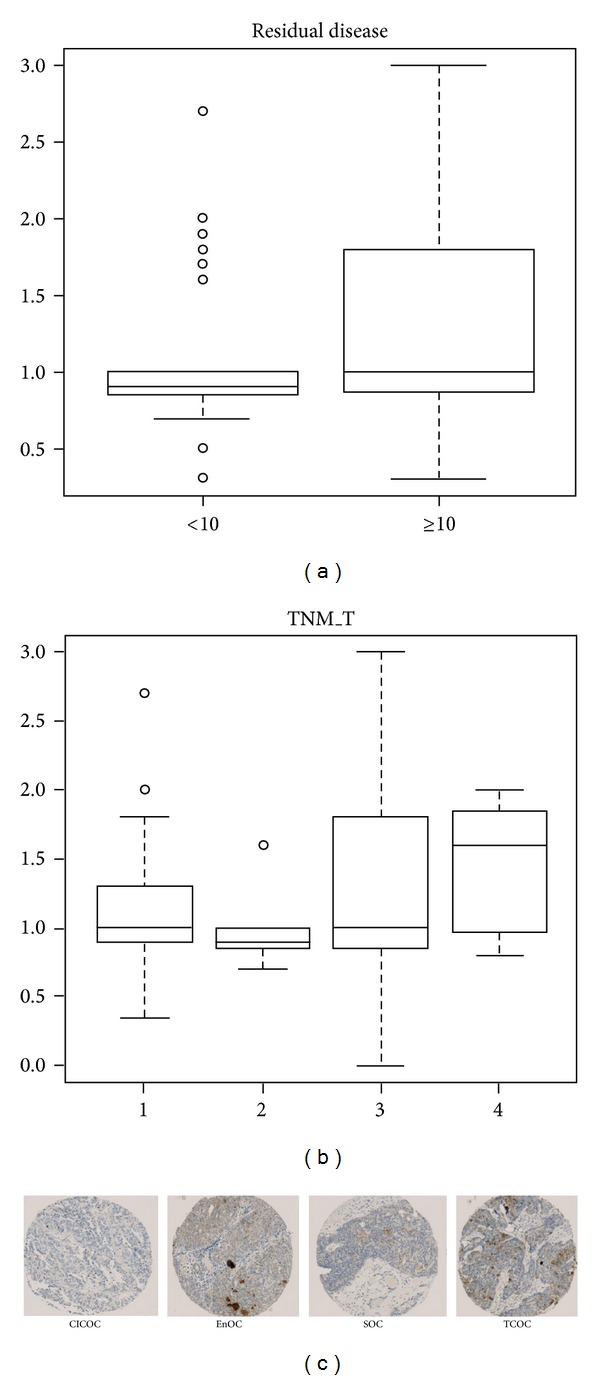
GAS6 expression level in relation to various clinicopathological parameters. (a) Ovarian cancers from patients with residual disease have significantly higher level of GAS6 expression. (b) Ovarian cancers from TMN T4 patients tended to have higher expression of GAS6. (c) GAS6 expression was similar across clear cell, endometrioid, serous, and transitional cell ovarian cancers as assessed by immunohistochemistry on tissue microarray. CICOC: clear cell ovarian cancer; EnOC: endometrioid ovarian cancer; SOC: serous ovarian cancer; TCOC: transitional cell ovarian cancer.

Within the cancer cohort (Table 1), GAS6 expression is significantly higher in tumours from patients with residual disease (defined as those with observable lesions greater than 10 mm in size) than those without (*P* = 0.0156) (Figure 2(a)). Similarly, under the TNM-M scoring system, tumours from T4 patients (*n* = 7) tended to have higher GAS6 expression compared to T1 (*n* = 32), T2 (*n* = 21), and T3 (*n* = 107) patients (patients with smaller tumours) (*P* = 0.07163) (Figure 2(b)). The expression level of GAS6 was not different across all histotypes investigated (Figure 2(c)). Similar observations were found with other clinicopathological parameters such as tumour stage, grade, presence of ascites at initial diagnosis, TMN-N (lymph node involvement) and TNM-M (metastases) status, and relapse status (data not shown). 

### 3.5. GAS6 Expression Is an Independent Predictor of Earlier Death

**Figure 3:**
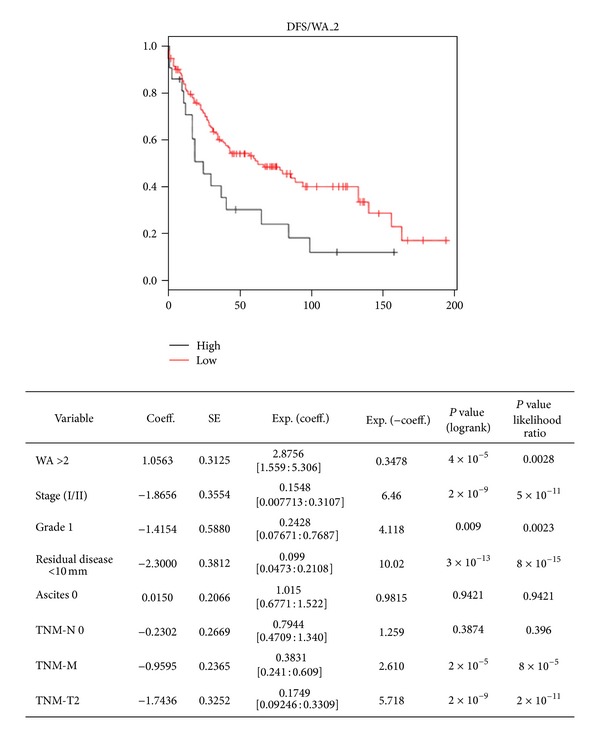
High expression of GAS6 correlates with shorter disease-free survival. The Kaplan-Meier curves of ovarian cancer patients stratified to high or low GAS6 expression (cutoff: WA of 2). Multivariate survival analysis indicates that high expression of GAS6 is an independent negative prognostic marker (*P* = 0.0028, likelihood ratio test).

To find associations between GAS6 expression levels and patient survival, univariate Cox proportional hazard models were constructed. High tumour expression of GAS6 (WA > 2) was associated with significantly shorter disease-free survival (*P* = 0.0004) (Figure 3). By multivariate analysis (proportional hazards model), GAS6 expression was still an independent negative prognostic factor (*P* = 0.0028, likelihood ratio). As expected, patients with earlier stage (FIGO I/II), grade 1, no residual disease, TMN-M 0, and TMN_T2 disease had significantly longer DFS than their counterparts (Figure 3). In terms of relapse-free survival, high GAS6 expression level had no effect (*P* = 0.2878) (data not shown). Again as expected, other clinicopathological parameters yielded significant *P* values (data not shown).

## 4. Discussion

Transcriptomic analysis of the cancer samples is frequently performed, often being a good starting point for the identification of novel biomarkers for a given cancer type. However, most transcriptomic studies to date have been performed on a single cohort and are often limited in sample size. Meta-analysis of combined transcriptomic microarray datasets is a powerful method to greatly increase the sensitivity in revealing biomarkers for disease and, potentially, with greater specificity. In order to identify new biomarkers for ovarian cancer based on survival, we performed meta-analysis of individual observations initially from four independent studies [6–9] and then after the retraction of the study by Dressman et al. [9] in 2012 from three independent studies. These analyses revealed GAS6 as our candidate of choice.

In our study, we found that GAS6 is overexpressed in ovarian cancer and therefore confirmed the findings of Sun et al. [12]. More importantly, however, we demonstrate that GAS6 is an independent predictor of earlier death. In addition, GAS6 expression level is higher in patients with greater tumour burden and/or with residual disease. These results suggest that both GAS6 transcript and protein may serve as biomarkers for ovarian cancer. In clinical practice, the staining of a tumour biopsy for GAS6 may be useful for cancer prognosis assessment and/or for disease progression monitoring.

Although the prognostic and diagnostic utility of biomarkers is important, the understanding of its pathophysiological role is also necessary as this knowledge may help in the design of a novel targeted therapy for the cancer. However, functional studies of GAS6 in ovarian cancer were beyond the scope of our study, but earlier studies have shown that GAS6 is a member of the vitamin K-dependent protein family [18]. It is structurally comprised of an N-terminal *γ*-carboxyglutamic (Gla) domain, four epidermal growth factor- (EGF-) like sequences, and a C-terminal composed of two globular laminin G-like (LG) domains [19]. GAS6 is a ligand for receptor tyrosine kinases (RTKs) of the TAM family: Tyro3, AXL, and MerTK, with binding affinities in the order of AXL > Sky > Mer [20]. The role of the GAS6/AXL axis in cancer is well documented. GAS6 is overexpressed in glioblastoma [10], gastric [11], and ovarian [12] cancers. Likewise, AXL is overexpressed in colon [21], thyroid [22], breast [23], renal cell [24], and ovarian [25] cancers. The latter study suggests that the GAS6/AXL axis may be involved in driving tumorigenesis of this cancer [25]. AXL protein expression was found to be significantly higher in ovarian carcinomas than in ovarian epithelium, and knockdown of AXL in SKOV3 cells also reduced the expression of MMP-1 and MMP-9, which contribute to tumour cell invasion. AXL knockdown also inhibited the size and the number of metastases in a xenogeneic mouse model of ovarian cancer metastasis. These experimental data together with our results that GAS6 overexpression is associated with shorter survival suggest that interventions which inhibit this pathway could have therapeutic potential. Since GAS6 is a secreted protein, a rational therapeutic approach is the development of an antibody against it. By reducing the amount of free GAS6 in serum and/or ascites, the direct growth-promoting effects of the GAS6/AXL may be transiently reduced. For this reason, an anti-GAS6 antibody may be employed as a useful adjunct therapy for ovarian cancer. There is also evidence in other cancer types that GAS6 may be involved in resistance to chemotherapy. It was shown that treatment of AXL-transfected U937 acute myeloid leukaemia cells with recombinant GAS6 resulted in resistance to doxorubicin, VP16, and cisplatin, an effect associated with increased expression of the antiapoptotic molecules Bcl-2 and Twist [26]. If the GAS6/AXL axis is also involved in chemoresistance in ovarian cancer, targeting this pathway may sensitise tumours to paclitaxel and carboplatin, the current mainstays of therapy, whilst also directly inhibiting tumour growth. Such studies are definitely warranted.

Although the majority of studies have shown that the GAS6/AXL axis helps drive tumorigenesis, there have been reports that suggest the contrary, which again highlights the notion that the function of GAS6 is highly context dependent. GAS6 was also shown to dose dependently inhibit the proliferation of the prostate cancer cell lines PC3 and DU145 [27], but the opposite effect was reported on the same cell lines by another group [28]. In a cohort of breast cancer patients, GAS6 expression positively correlated with favourable prognostic variables such as lymph node negativity, smaller tumour size, and low Nottingham prognostic index score [29]. Therefore, from a therapeutic point of view, more robust studies on the function of GAS6 in ovarian cancer are required.

## Figures and Tables

**Table 1:** Clinicopathological data for nonmucinous EOC cohort (*n* = 172).

Variable	Number	%
Age at diagnosis		
>60	97	56.4
<60	73	43.6
FIGO stage		
I	32	18.6
II	17	9.9
III	72	41.9
IV	43	25.0
NA	8	4.6
TNM-T		
1	32	18.6
2	21	12.2
3	107	62.2
4	7	4.1
NA	5	2.9
TNM-N		
0	96	55.8
1	41	23.8
NA	35	20.4
TNM-M		
0	101	58.7
1	45	26.2
NA	26	15.1
Grade		
1	15	8.7
2	58	33.7
3	98	57.0
NA	1	0.6
Histological type		
Serous (SOC)	84	48.8
Endometrioid (EnOC)	39	22.7
Clear cell (ClCOC)	42	24.4
Transitional cell (TCOC)	7	4.1
Ascites at primary diagnosis		
Yes	89	51.7
No	83	48.3
Residual disease		
≥10 mm	84	48.8
≤10 mm	49	28.5
NA	32	22.7
Primary cancer origin		
Both ovaries	80	46.5
Left ovary	43	25.0
Right ovary	40	23.2
NA	9	5.3
Performance status		
Healthy	77	44.8
Systematic disease	55	31.9
Severely sick	1	0.6
NA	39	22.7
With cancer family history CA125 (preoperative)		
Yes	40	23.3
≥35 U/mL	75	43.6
≤35 U/mL	10	5.8
NA	87	50.6
Relapse		
Yes	140	81.4
No	32	18.6
Platinum chemotherapy		
Sensitive	45	26.2
Resistant	93	54.0
NA	34	19.8
Outcome		
Death related to malignancy	88	51.2
Unrelated, no disease	1	0.6
NA	83	48.2
Progressive disease (months)		
Yes	36	20.9
No	136	79.1

**Table 2:** Top 34 overexpressed genes obtained by meta-analysis.

Gene	SD	Comb. *Z*
*R3HDM1 *	0.3	3.45
*ATR *	0.3	3.23
*UVRAG *	0.3	3.16
*TARBP1 *	0.31	3.01
*PCNA *	0.39	3.09
*ZNF184 *	0.44	3.26
*DSC2 *	0.45	3.75
*CDH6 *	0.55	3.17
*NUP205 *	0.59	3.07
*NEK2 *	0.63	3.05
*EYA2 *	0.71	3.78
*E2F3 *	0.72	3.17
*SOX9 *	0.75	3.56
*PEA15 *	0.86	3.82
*SPOCK2 *	0.88	3.5
*BTG3 *	0.89	3.25
*FDPS *	0.93	3.68
***GAS6***	**0.95**	**3.10**
*DDR1 *	0.96	3.93
*HLTF *	0.97	3.08
*PRDX6 *	0.98	3.05
*IGFBP2 *	1.07	3.48
*CELSR2 *	1.13	3.1
*ASNS *	1.17	3.49
*SMG7 *	1.25	3.46
*CD47 *	1.29	3.89
*CXADR *	1.3	5.64
*CRIP2 *	1.3	3.74
*GUK1 *	1.38	3.81
*BMI1 *	1.5	3.37
*MEIS1 *	1.64	4.45
*WFDC2 *	1.66	5.88
*RBM38 *	1.91	3.18
*SCNN1A *	1.94	3.77
